# Case Report: Double Visualization Intubation Strategy for Patients With Ankylosing Spondylitis

**DOI:** 10.3389/fmed.2022.659624

**Published:** 2022-03-18

**Authors:** Wei-can Chen, Shu Lin, He-fan He

**Affiliations:** ^1^Department of Anaesthesiology, The Second Affiliated Hospital, Fujian Medical University, Quanzhou, China; ^2^Centre of Neurological and Metabolic Research, The Second Affiliated Hospital, Fujian Medical University, Quanzhou, China; ^3^Diabetes and Metabolism Division, Garvan Institute of Medical Research, Sydney, NSW, Australia

**Keywords:** ankylosing spondylitis, intubation, difficult airway, visualization, case report

## Abstract

**Background:**

Ankylosing spondylitis is an autoimmune disease involving the axial bone. Because it leads to rigidity of the spine and joints, especially when involving the cervical spine, it will cause a difficult airway, creating a major challenge for airway management. Herein, we report presents a double visual intubation strategy for severe ankylosing spondylitis patients who are difficult to intubate with a video laryngoscope.

**Case Presentation:**

A 31-year-old patient with severe ankylosing spondylitis has a seriously restricted neck movement that makes it hard to insert a tracheal tube using only a video laryngoscope. With the aid of video laryngoscope, we then guided the endotracheal intubation using a lighted stylet. Eventually, the oropharynx was opened sufficiently so that the tracheal tube could be rapidly introduced below the epiglottis and entered the glottis.

**Conclusion:**

In conclusion, the video laryngoscope is a viable operation to assist lighted stylet guided endotracheal intubation in severe ankylosing spondylitis patients after video laryngoscope intubation failure.

## Introduction

Ankylosing spondylitis (AS) has a strong association with HLA-B27 genotypes and manifests as a chronic autoimmune disease with axial bone inflammation (1). The disease manifests as fibrosis and ossification of the large joints of the extremities and the annulus fibrosus of the intervertebral disks and their nearby connective tissue, which in turn leads to spinal and joint ankylosis (2). When AS involves the cervical spine, it may cause difficult airways, especially when temporomandibular is involved. This poses a major challenge to the airway management of the anesthesiologist (3).

We present a double visual intubation strategy for severe AS patients who are difficult to intubate with a video laryngoscope (VL, UE Inc., Taizhou, ZheJiang). The patient consented to be in this report.

## Case Presentation

**Figure 1:**
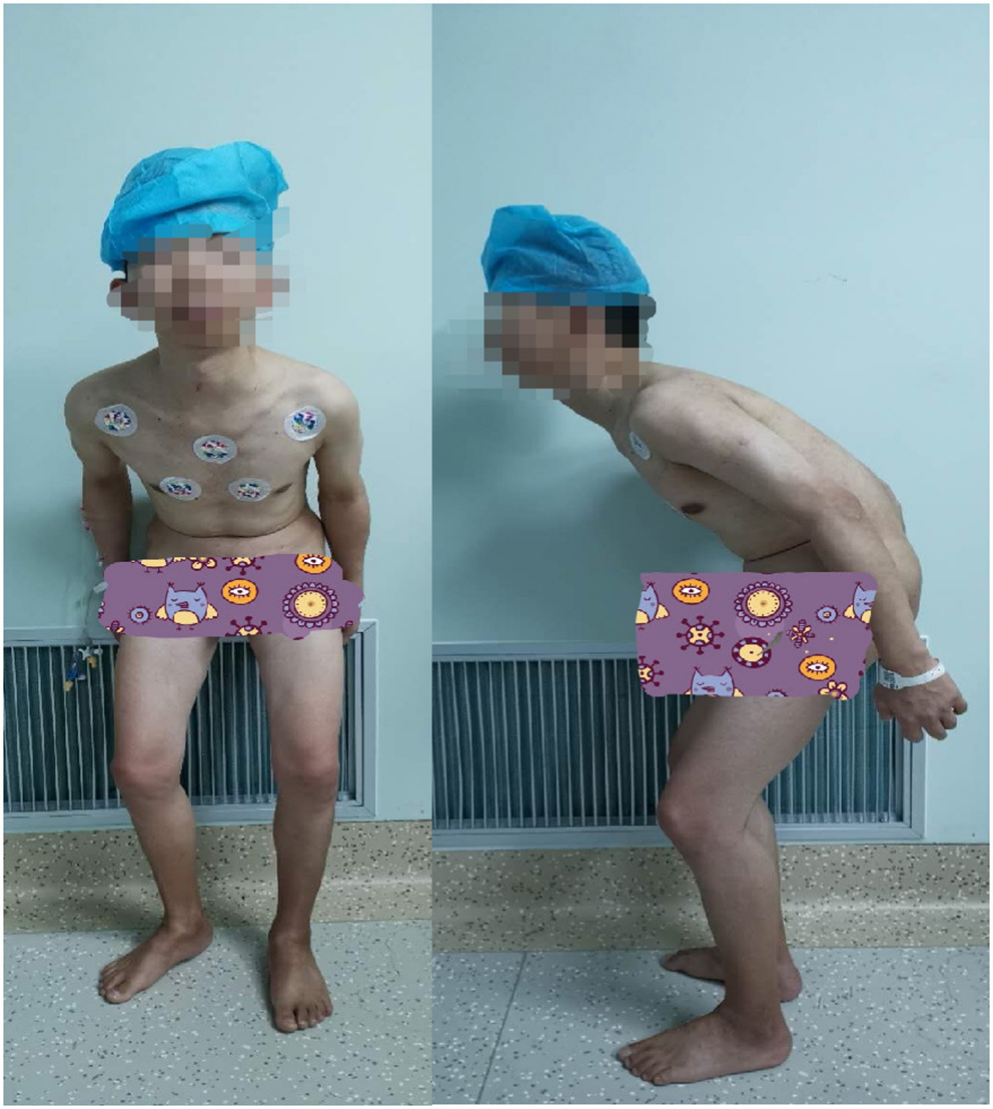
Front and side views of the patient.

**Figure 2:**
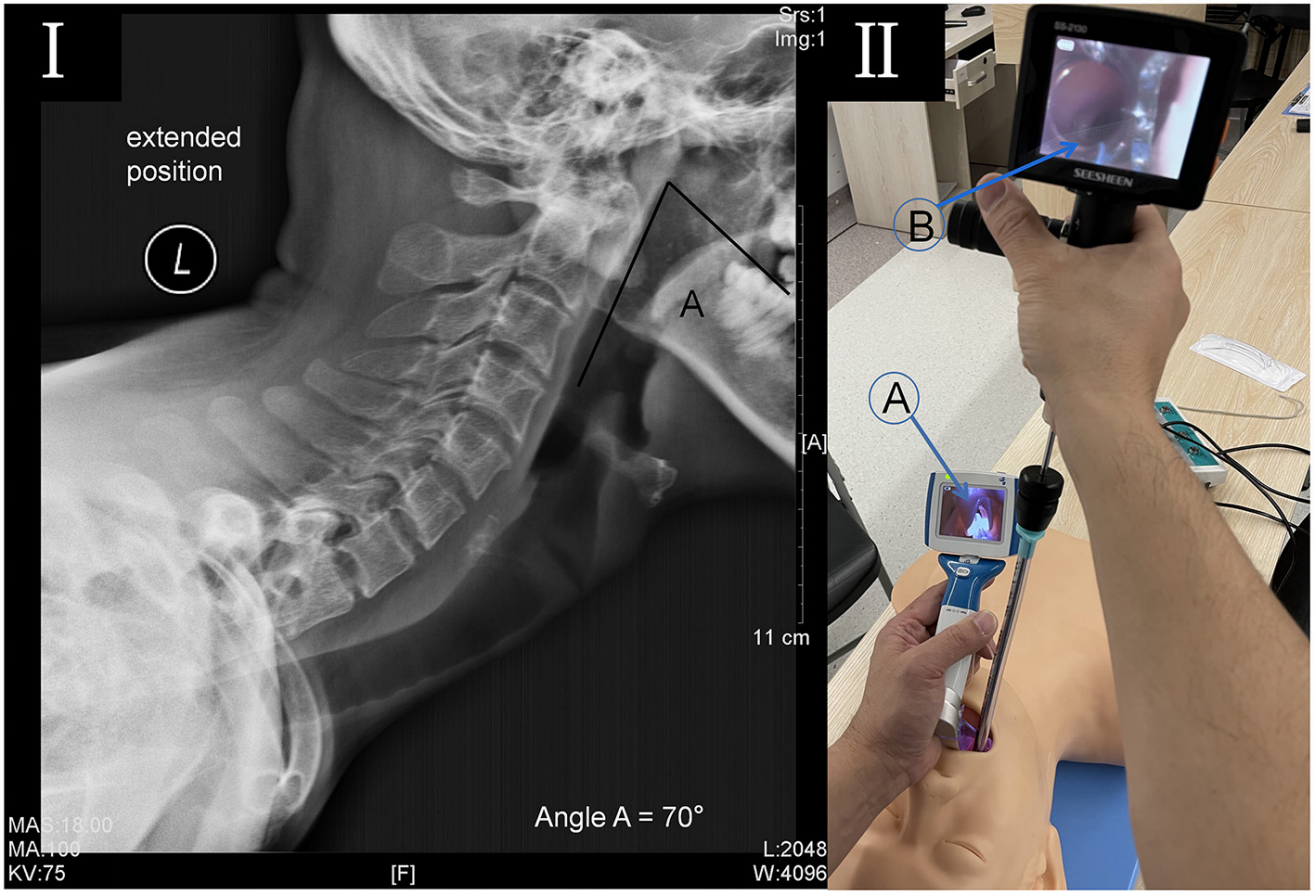
**(I)** Lateral radiograph of the cervical spine in full extension. The photograph demonstrates that hyperostosis was seen at the cervical vertebral body margin, and the oropharyngeal axis (angle a) was 70°. **(II)** A double visualized intubation on a mannequin. (A) The video laryngoscope opens the oropharynx to show the epiglottis; (B) The lighted stylet uncovers the epiglottis to expose the glottis and guide the tracheal tube into the trachea.

A 31-year-old male patient (height 131 cm, weight 31 kg, ASA III, Mallampati III.), with severe AS, underwent lumbar orthopedic surgery. Physical examination revealed a deformity of the spine, with the spine tilted forward and fixed (Figure 1), posterior extension and lateral bending limitation, schooner test was <4 cm, pelvic lateral pressure test and “4” test were positive. Biochemical examination showed WBC 19.05 × 10^9^/L, NE 92.1%, alanine aminotransferase 6 IU/L, aspartate aminotransferase 9 U/L and HLA-B27 positive, and thoracic MRI showed thoracic and lumbar kyphosis changes, and the upper and lower margins of the vertebral bodies were straight, which conformed to the changes of ankylosing spondylitis. His mouth opening was 30 mm and thyromental distance was 55 mm. The cervical spine was solidified with maximum flexion and extension of 25°, and an oropharyngeal axis angle of 70° (Figure 2I).

The patient's ECG, SpO_2_, blood pressure, and end-tidal CO_2_ were routinely monitored. 0.5 mg penehyclidine was administrated to reduce airway secretions and 2% lidocaine was used for pharyngeal surface anesthesia. We administered midazolam 3 mg and sufentanil 10 μg for conscious sedation with preserved spontaneous breathing and then awake intubation with double visualization. Patients with AS usually can't lie down, so we intubated in a sitting position. Neither the VL nor the introduction tool effectively revealed the epiglottis due to constrained neck movement. As a result, the endotracheal tube could not be inserted successfully. With the aid of VL, we guided the endotracheal intubation using a rigid lighted stylet (LS). The oropharynx was opened sufficiently so that the tracheal tube could be rapidly introduced below the epiglottis and entered the glottis. After intubation, connect the anesthesia threaded tube to listen to the breathing sounds of both lungs and give deep anesthesia and muscle relaxants symmetrically. Sufentanil 30 μg, cis-atracuramine 5 mg/h and 2% sevoflurane inhalation were used to maintain anesthesia. The operation lasted for 3 h and the bleeding was 100 ml. The operation proceeded uneventfully. After the operation, the patient was fully awake and had enough ventilation, swallowing, and cough reflexes recovered. Use a tube exchanger for extubation after sufficient sputum suction. The patient recovered well without postoperative complications.

## Discussion

Early studies (4, 5) have revealed that VL is a beneficial device to improve visualization of the larynx and expedite endotracheal intubation in anesthetized patients with AS. Indeed, for novice anesthesiologists, visualization intubation strategy is especially beneficial (6). In this study, several patients who could not be intubated using a VL had a thyromental distance of <65 mm. The reason for this is perhaps due to the restriction of the temporomandibular extension in the patients and the resistance that may be encountered during tracheal tube advancement.

In our case, despite the use of VL, laryngeal tissue was unable to be revealed. This may be due to the limited distance between the base of the tongue and the soft palate, and the patient's oropharyngeal axis angle <90°. This results in a situation wherein the laryngeal mask is also difficult to insert (7), resulting in the VL not being able to be entirely placed correctly. In addition, the guidance of the introducer tool also encountered considerable resistance, making it impossible to insert the tracheal tube successfully. The death of retropharyngeal abscess due to multiple attempts at blind intubation has been reported (8). Therefore, when the VL shows the oropharyngeal space and the epiglottis, it is considered safer to expose the glottis and guide the trachea into the trachea with LS (Figure 2II).

Awake fiberoptic bronchoscopy is another safe option for patients with AS (3). This intubation method has low risk and high success rate and should be given priority. However, some patients are incapable of enduring the procedure and refuse conscious intubation. In our case, adequate sedation was maintained while spontaneous breathing was maintained, and it should be said that tracheal intubation *via* nasal fibrobronchoscopy *via* VL was also a good option. But for patients with nasal disease or without fibrosoft mirrors, it is also a good idea to intubate *via* the oropharynx using VL combined with LS. In addition, for patients who refuse conscious intubation, laryngeal mask ventilation may be another option (9). However, the laryngeal mask cannot provide stable airway management, especially for operations that require the patient to be in a specific position, such as the lumbar spine orthopedic surgery in this case. Nevertheless, based on our experience, we believe that the laryngeal mask is a suitable alternative for airway management in the cases of intubation failure after anesthesia. It should be noted that when the oropharyngeal axis angle is <90°, the laryngeal mask is not easy to position correctly due to the anterior section of the cuff having a tendency to fold over (7).

## Conclusion

VL combined with LS is beneficial for AS patients with difficult intubation. Primarily, VL entirely unfolds the oropharynx cavity and provides good visualization of the pharynx (Figure 2IIA). Furthermore, with VL's support, the LS swiftly inserts under the epiglottis, to expose the glottis and guide the tracheal tube's insertion (Figure 2IIB). Assuredly, this procedure does not require lifting the jaw bone to reveal the pharynx, which is effective and safe for patients with restricted neck flexibility. In conclusion, VL is a viable operation to assist LS guided endotracheal intubation in severe AS patients after VL intubation failure.

## Data Availability Statement

The original contributions presented in the study are included in the article/supplementary material, further inquiries can be directed to the corresponding author/s.

## Ethics Statement

Written informed consent was obtained from the individual(s) for the publication of any potentially identifiable images or data included in this article.

## Author Contributions

W-cC, H-fH, and SL contributed to the conception and design of the review. W-cC drafted and finalized the manuscript. H-fH and SL revised the manuscript and provided critical advice on the content of the manuscript. All authors approved the submitted version.

## Conflict of Interest

The authors declare that the research was conducted in the absence of any commercial or financial relationships that could be construed as a potential conflict of interest.

## Publisher's Note

All claims expressed in this article are solely those of the authors and do not necessarily represent those of their affiliated organizations, or those of the publisher, the editors and the reviewers. Any product that may be evaluated in this article, or claim that may be made by its manufacturer, is not guaranteed or endorsed by the publisher.

## References

[1] Voruganti A Bowness P. New developments in our understanding of ankylosing spondylitis pathogenesis. Immunology. (2020) 161:94–102. 10.1111/imm.13242 32696457 PMC7496782

[2] Taurog JD Chhabra A Colbert RA. Ankylosing spondylitis and axial spondyloarthritis. N Engl J Med. (2016) 374:2563–74. 10.1056/NEJMra1406182 27355535

[3] Woodward LJ Kam PCA. Ankylosing spondylitis: recent developments and anaesthetic implications. Anaesthesia. (2009) 64:540–8. 10.1111/j.1365-2044.2008.05794.x 19413825

[4] Lili X Zhiyong H Jianjun S. A comparison of the GlideScope with the Macintosh laryngoscope for nasotracheal intubation in patients with ankylosing spondylitis. J Neurosurg Anesth. (2014) 26:27–31. 10.1097/ANA.0b013e31829a0491 23764717

[5] Lewis SR Butler AR Parker J Cook TM Smith AF. Videolaryngoscopy versus direct laryngoscopy for adult patients requiring tracheal intubation. Cochrane Database Syst Rev. (2016) 11:D11136. 10.1002/14651858.CD011136.pub2 27844477 PMC6472630

[6] Soleimanpour H Panahi JR Mahmoodpoor A Ghafouri RR. Digital intubation training in residency program as an alternative method in airway management. Pak J Med Sci. (2011) 27:401–4.

[7] Chiu P Cheng K Tseng K Shih C Chen M. Fibreoptic bronchoscopy to facilitate ProSeal laryngeal mask airway insertion in a patient with ankylosing spondylitis. Anaesthesia. (2011) 66:138–9. 10.1111/j.1365-2044.2010.06594.x 21254993

[8] Hill CM. Death following dental clearance in a patient suffering from ankylosing spondylitis - a case report with discussion on management of such problems. Br J Oral Sur. (1980) 18:73–6. 10.1016/0007-117X(80)90054-2 6462168

[9] Herman AG Mahla ME. Awake intubating laryngeal mask airway placement in a morbidly obese patient with ankylosing spondylitis and unstable thoracic spine. J Clin Anesth. (2016) 32:62–4. 10.1016/j.jclinane.2015.12.021 27290947

